# Gastric Schwannoma: A Rare Subepithelial Lesion of the Stomach

**DOI:** 10.7759/cureus.44577

**Published:** 2023-09-02

**Authors:** Sameeta Kumari, Muhammad ibrahim Saeed, Adeel Urrehman

**Affiliations:** 1 Gastroenterology, Aga Khan University Medical College, Karachi, PAK; 2 Gastroenterology, Aga Khan University Hospital, Karachi, PAK; 3 Medicine, Aga Khan University Hospital, Karachi, PAK

**Keywords:** surgery, immune staining, endoscopic ultrasound, gastric schwannoma, subepithelial lesions

## Abstract

Gastric schwannomas (GS) are rare, slow-growing, benign subepithelial (SE) lesions of the stomach. These are difficult to differentiate preoperatively from other types of SE lesions, as the gross appearance and clinical presentation are very similar especially the gastrointestinal stromal tumors (GISTs), which are the most common SE lesions of the stomach. We present the case of a 35-year-old Asian man with a one-month history of intermittent, right, upper quadrant pain and hematemesis. Preoperatively, intravenous contrast-enhanced computer tomography scan (CECT) showed a well-circumscribed lesion in the upper abdomen closer to the stomach’s lesser curvature. Endoscopic ultrasound revealed an ulcerated subepithelial lesion and subsequent biopsy with a 22-gauge fine-needle biopsy (22G FNB) needle confirmed the diagnosis of GS. The patient underwent distal gastrectomy and a Bilroth 1 procedure. The postoperative biopsy was consistent with the initial one, therefore supporting the diagnosis of gastric schwannoma. Postoperatively, the recovery remained uneventful.

## Introduction

Gastric schwannomas are mesenchymal tumors derived from the nerve plexus of the gastrointestinal (GI) tract; they are one of the several types of spindle cell tumors that can be encountered in the GI tract. The other spindle cell tumors include GISTs, leiomyosarcoma, inflammatory fibroid polyps, solitary fibrous tumors, and inflammatory myeloblastic tumors [1]. Among these, GISTs are the most common tumors, predominantly occurring in the stomach (60% to 70%) [1,2]. On the contrary, schwannomas rarely involve the gastrointestinal tract; they represent only 0.2% of all gastric tumors and 4% of all benign gastric neoplasms [3]. Although gross appearance and clinical presentation are similar to GISTs, schwannomas have different therapeutic options and prognoses. Moreover, they have a slower multiplication rate and thus have a better prognosis [4].

## Case presentation

A 35-year-old Asian man presented in an outpatient gastroenterology clinic in April 2022 with no known comorbidities. He had a one-month history of intermittent right upper quadrant pain and hematemesis. Initial labs included a normocytic normochromic profile on a complete blood count sample with a hemoglobin of 12.8 g/dl (normal 14-16 g/dl) and a hematocrit of 38.5 % (normal 41-50%). His prothrombin time and international normalized ratio (INR) were within normal limits. However, he had a history of three units of packed red blood cell transfusions, as his hemoglobin levels had dropped to 6 g/dl one month ago.

**Figure 1:**
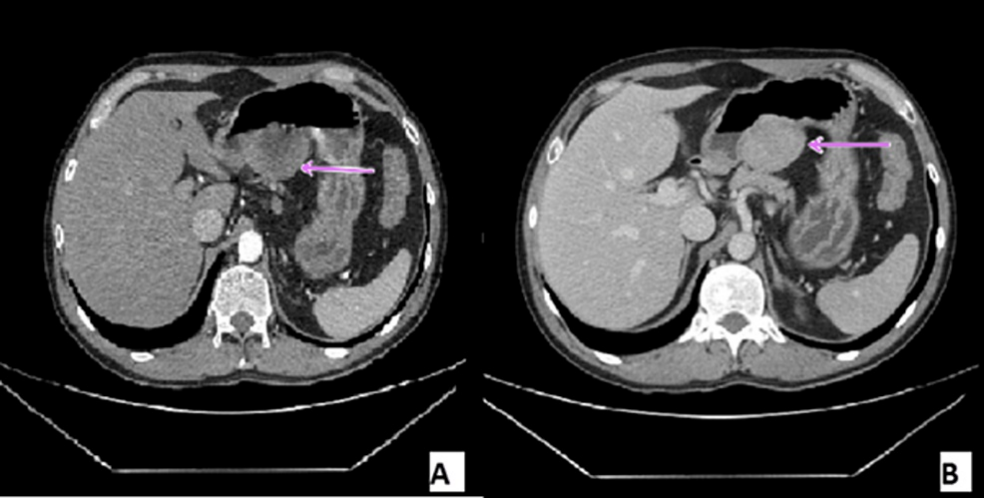
Contrast-enhanced CT scan shows a round homogeneous lesion (arrow) arising from the lesser curvature of the stomach showing homogenous enhancement in the venous phase A - arterial phase image, B - venous phase image

**Figure 2:**
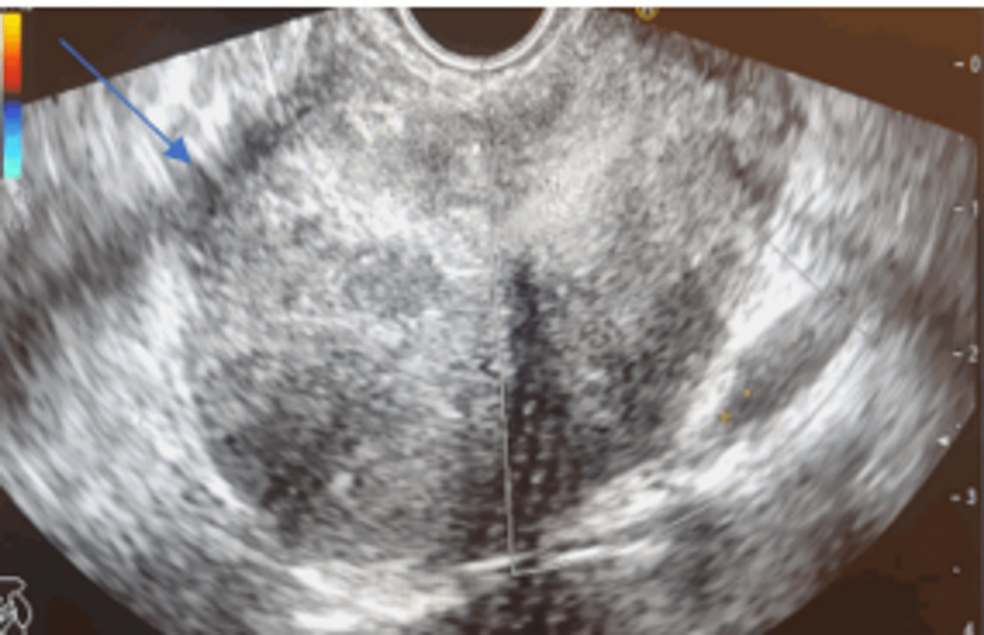
EUS image shows a well-defined gastric mass (5x5 cm) with irregular borders and heterogenous echogenicity arising from the fourth layer (arrow) EUS - endoscopic ultrasound

Therefore, imaging was advised and CECT abdomen was performed. It suggested a well-circumscribed, lobulated, enhancing lesion in the upper abdomen closer to the lesser curvature of the stomach and the pancreatic body (Figure 1). Endoscopic ultrasound (EUS) confirmed the diagnosis, revealing an ulcerated subepithelial mass measuring approximately 5x5 cm in maximum dimension with perilesional small lymph nodes (Figure 2).

**Figure 3:**
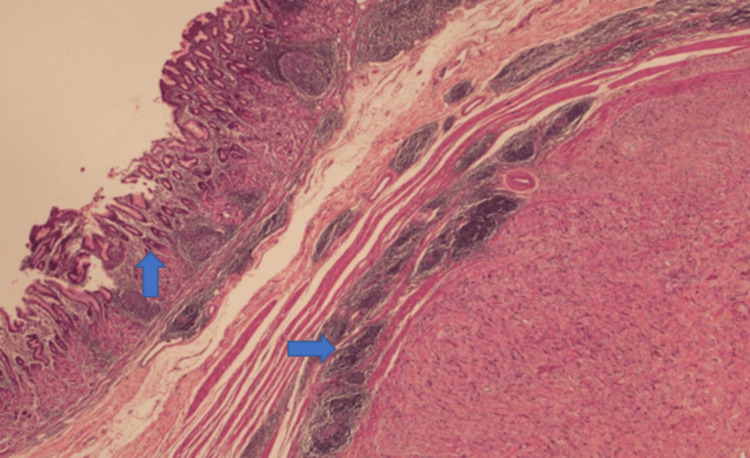
The resected gastric mass showing a circumscribed spindle cell tumor with a lymphoid cuff at the periphery (→) and intact mucosa on the surface (↑)

**Figure 4:**
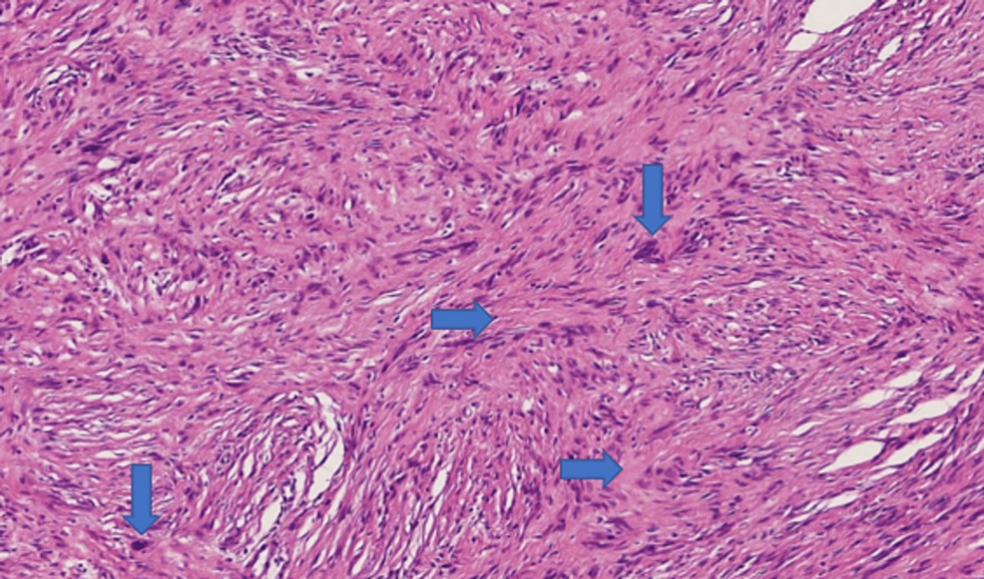
The resected gastric mass showing a spindle cell tumor with nuclear palisading forming nuclear-free zones or Verocay bodies (→) and degenerative atypia (↓) without any significant mitoses

**Figure 5:**
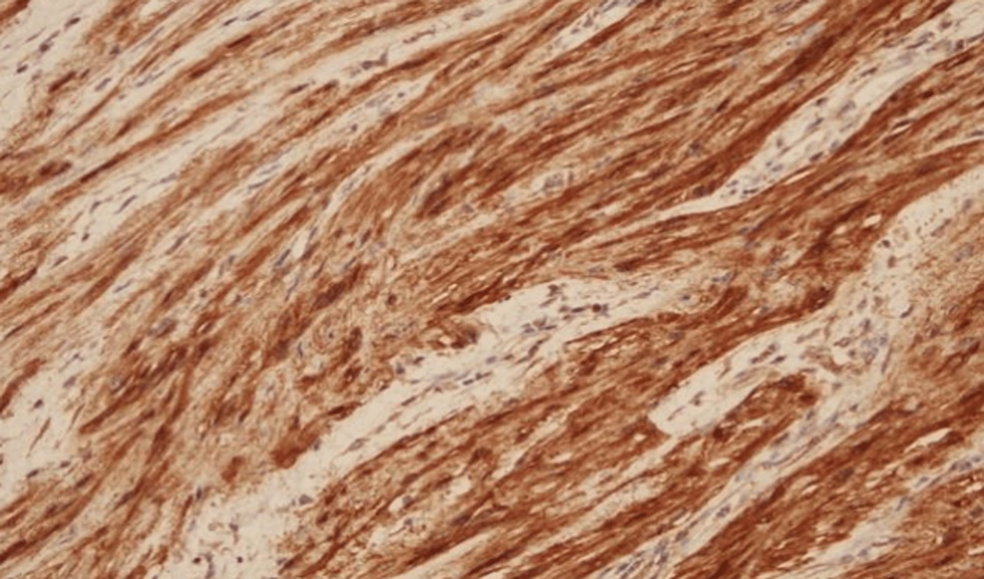
The morphological findings with immunohistochemical strong positivity for S100 are consistent with the diagnosis of schwannoma

A biopsy was performed using the 22G fine-needle biopsy (FNB) needle. The microscopic examination of the resected stomach mass confirmed linear cores of spindle cells arranged in sheets with abundant collagenized stroma (Figure 3). Moreover, there were lymphoid aggregates in some areas. However, no mitotic activity was seen (Figure 4). Immunohistochemical stains led to the diagnosis of a gastric schwannoma. The tumor was positive for S100 (Figure 5) and SOX-10, and negative for CD117, DOG 1, CKAE1/AE3, CD34, h-caldesmon, desmin, myogenin, ALK protein, HMB45, and beta catenin. It also stained low for Ki67.

Subsequently, this patient was admitted under the surgery team and underwent distal gastrectomy and the Bilroth 1 procedure. The intraoperative findings included a 4x4 cm tumor at the posterior surface of the left curvature of the stomach with no gross ascites, serosal, peritoneal, or hepatic metastasis. The postoperative time was uneventful, and the patient was discharged on the sixth postoperative day. During his follow-up after one month, he was absolutely asymptomatic. The final biopsy confirmed the diagnosis of a gastric schwannoma, as the histopathology report and immunohistochemical stains remained the same on testing.

## Discussion

Gastrointestinal tumors are unique mesenchymal neoplasms; they display salient variations from the typical schwannomas, occurring in the soft tissues and central nervous system [5]. Furthermore, the incidence of gastric schwannomas is nearly identical in both males and females, with a mean age commonly falling within the sixth to seventh decade of life [6]. They are most commonly found in the gastric body (73.7%), followed by the gastric antrum and gastric fundus (15.8% and 10.5%, respectively) [7]. On CT scans, they are well-defined solid masses, and homogenous enhancement is seen in more than 90% of the cases [7]. The CT scan findings are consistent with our case presentation, as the report suggested a well-circumscribed enhancing lesion. However, the patient was of a younger age compared to the mean.

Grossly, schwannomas appear as rubbery, uniform, homogenous, unencapsulated, and yellow-white to tan circular nodules [8]. Histologically, spindle cell schwannoma is the most frequently encountered type, although there are some other variants, including the epithelioid and plexiform ones [8]. The cytoplasm of these cells is mostly eosinophilic and lacks evident coarse fibrillar material or discernible cell walls. In addition, the nuclei of these cells are generally thinner in comparison to the nuclei of smooth muscle cells [6]. Moreover, most schwannomas typically have no mitotic activity. Most of the features are similar to our case presentation.

Immunohistochemically, gastric schwannomas have some common features. These include positive S100 protein and SOX 10 and negative or only locally positive CD34 and DOG-1 [9]. All of these are consistent with our patient’s biopsy report.

Management includes endoscopic and surgical resection. Endoscopic resection is preferred when the lesion is small (less than 2 cm), arises from the submucosa or muscularis propria, and is located on the greater curvature or anterior wall of the stomach. Other features that favor endoscopic therapy include non-ulcerated and protuberant morphology [9]. Lesions that are larger in size, have ulcerated and extraluminal growth, and are located on the lesser curvature are difficult to manage endoscopically, and in these cases, surgical resection is the optimal treatment [10]. Our patient underwent surgical resection because of the large size of the lesion and its location near the lesser curvature.

## Conclusions

In conclusion, a gastric schwannoma is a slow-paced, rare SE mesenchymal tumor of the gastrointestinal tract; its gross appearance, radiological features, and clinical presentation resemble GISTs, which makes it challenging to diagnose it preoperatively. Despite the similarity in clinical and radiological manifestations, the prognosis between the two lesions is different, as GISTs can progress to malignant lesions in some cases while schwannomas are almost always benign. Hence, it is imperative to include it in the differentials of subepithelial lesions by confirming the diagnosis with biopsy and immunohistochemical stains.
